# The Pattern and Distribution of Deleterious Mutations in Maize

**DOI:** 10.1534/g3.113.008870

**Published:** 2013-11-26

**Authors:** Sofiane Mezmouk, Jeffrey Ross-Ibarra

**Affiliations:** * Department of Plant Sciences, University of California–Davis, Davis, California 95616; † Center for Population Biology and Genome Center, University of California–Davis, Davis, California 95616

**Keywords:** Deleterious mutations, GBS SNPs, Heterosis, Maize, Quantitative traits

## Abstract

Most nonsynonymous mutations are thought to be deleterious because of their effect on protein sequence and are expected to be removed or kept at low frequency by the action of natural selection. Nonetheless, the effect of positive selection on linked sites or drift in small or inbred populations may also impact the evolution of deleterious alleles. Despite their potential to affect complex trait phenotypes, deleterious alleles are difficult to study precisely because they are often at low frequency. Here, we made use of genome-wide genotyping data to characterize deleterious variants in a large panel of maize inbred lines. We show that, despite small effective population sizes and inbreeding, most putatively deleterious SNPs are indeed at low frequencies within individual genetic groups. We find that genes associated with a number of complex traits are enriched for deleterious variants. Together, these data are consistent with the dominance model of heterosis, in which complementation of numerous low-frequency, weak deleterious variants contribute to hybrid vigor.

The effect of new mutations on organismal fitness is not well understood, but both theoretical considerations (Fisher 1930) and empirical estimates (Joseph and Hall 2004) suggest that most new mutations are deleterious and that only a small minority are beneficial. Strongly deleterious mutations are expected to be kept at low frequencies by natural selection, whereas weakly deleterious alleles may be effectively neutral (Ohta 1973; Kimura 1983) and subject to the effects of genetic drift (Lynch and Gabriel 1990; Lande 1994; Whitlock *et al.* 2003). In addition to selection and drift, a number of other factors such as mating system and recombination rate also impact the evolution of deleterious alleles. Selfing species and inbreeding within populations will expose lethal mutations to selection faster than in an outcrossing population (Wang *et al.* 1999; Glémin *et al.* 2003). Moreover, in genomic regions with low levels of recombination, selection against deleterious mutations will be less effective (Charlesworth *et al.* 1993), and the potential exists for deleterious mutations to rise to high frequency due to the effects of linked selection on beneficial mutations (Felsenstein 1974; Hill and Robertson 1966; Chun and Fay 2011).

Deleterious alleles may play an important functional role in affecting the phenotype of traits of interest, and complementation between haplotypes carrying different deleterious alleles may explain much of the observation of hybrid vigor or heterosis (Charlesworth and Willis 2009). In studies of human disease, a significant correlation was observed between the deleterious predictions of single-nucleotide polymorphisms (SNPs) and their association with cancer (Zhu *et al.* 2004); predicted rare, deleterious SNPs also were shown to be involved in common diseases (Cohen *et al.* 2004; Smigrodzki *et al.* 2004). Furthermore, rare, deleterious SNPs have gained interest as the result of their potential role in explaining quantitative trait variation (Gibson 2012), especially in populations that have experienced recent growth (Lohmueller 2013).

Evaluating the abundance and frequency of deleterious mutations is thus of considerable interest and has been investigated in a wide range of species. These analyses have varied in terms of the percentage of nonsynonymous sites estimated to be deleterious, from 3% in bacterial populations (Hughes 2005) to 80% in the human genome (Fay *et al.* 2001). They have also shown that recently bottlenecked populations may have a higher abundance of deleterious sites and that heterozygosity at deleterious SNPs is lower than at synonymous SNPs (Lohmueller *et al.* 2008). In plants, Gossmann *et al.* (2010) found that most new mutations in plants are strongly deleterious, with only 25% acting as effectively neutral. Cao *et al.* (2011) show that the abundance of deleterious variants correlates with effective population size in *Arabidopsis thaliana*, and a demographic bottleneck appears to have relaxed purifying selection in *Capsella rubella* (Brandvain *et al.* 2013). Other analyses of purifying selection in plants have implicated a role for environmental differences (Tellier *et al.* 2011) and identified differences among genes based on their level of expression (Paape *et al.* 2013). In natural populations of *Arabidopsis thaliana*, selection appears to act to maintain variants that are locally adaptive but deleterious elsewhere (Fournier-Level *et al.* 2011), whereas positive selection on domestication genes may have increased the abundance of deleterious variants in domesticated genomes such as rice (Günther and Schmid 2010; Lu *et al.* 2006). Although these studies have provided insight into the evolutionary fate of deleterious mutations, we still understand relatively little about the role of deleterious variants in effecting phenotypic traits.

Maize (*Zea mays*) is a economically important cereal worldwide, with the highest yield and one of largest cultivated areas (FAO statistics, http://faostat.fao.org); it is also an important model for basic and applied research (Strable and Scanlon 2009). Maize traditionally was cultivated in open pollinated populations (landraces) but, after the first documented observations of hybrid vigor in this species (East 1907−1908; Shull 1908), inbred lines were developed and structured into heterotic groups that maximize intergroup combining ability. The transition from heterozygous populations to strongly structured heterotic groups of inbred lines makes maize of interest for analyzing the distribution and frequency of deleterious mutations. Furthermore, high observed values of hybrid vigor or heterosis in maize hybrids makes it an excellent system for studying the effects of deleterious mutations and their contribution to heterosis. The dominance model of heterosis posits that inbred lines are homozygous for a number of recessive deleterious alleles and that crosses between inbreds carrying different complements of deleterious alleles will result in heterozygous progeny with higher fitness than either parent.

The aim of the current study was to (1) carry out a genome-wide scan for deleterious mutations in a maize diversity panel, (2) analyze their distribution across the genome and within different genetic groups, and (3) test for enrichment of deleterious loci in the results of genome-wide association mapping. High-density SNPs and phenotypic data available for a large sample of inbred lines and hybrids were used to address these questions. Our results showed that maize inbred lines are segregating for a large number of predicted deleterious variants (20−40% of protein coding SNPs were predicted to have a deleterious allele), and that these alleles are generally at very low frequencies with few fixed differences observed among different genetic groups. Genome-wide association analysis of hybrid vigor finds little evidence for enrichment of individual deleterious SNPs, but significant enrichment for genes containing deleterious SNPs, suggesting a meaningful role for dominance and complementation in explaining observations of hybrid vigor.

## Materials and methods

### Plant material and phenotypic data

We used phenotypic data (File S3) published in Flint-Garcia *et al.* (2005) for 247 maize inbred lines (see Supporting Information, File S1 for a list of inbred lines). Each inbred line was crossed to the stiff-stalk inbred B73 (population A) and both the inbred lines and their B73-hybrids were evaluated in 2003, in adjacent blocks within three environments with a single replicate in each (Flint-Garcia *et al.* 2009). A subset of 102 inbreds were additionally crossed to both B73 (population B1) and Mo17 (population B2); both inbred lines and hybrids were evaluated in a single environment in 2006 (Flint-Garcia *et al.* 2009). Table S1 lists the analyzed traits that are detailed in Flint-Garcia *et al.* (2009).

The panel structure was previously analyzed (Flint-Garcia *et al.* 2005), and inbred lines were attributed to the following subpopulations: stiff-stalk (27 inbred lines), non-stiff stalk (90 inbred lines), tropicals (60 inbred lines), popcorns (eight inbred lines), sweet (six inbred lines), and mixed (56 inbred lines). For the main temperate inbred lines, these subpopulations correspond to the different heterotic groups.

### Genotypic data

We made use of genotypic data from Larsson *et al.* (2013) for the full set of 247 lines (File S4). The latter were genotyped using the genotyping-by-sequencing approach (GBS; Elshire *et al.* 2011), resulting in a total of 437,650 partially imputed SNPs. Of these SNPs, 127,994 mapped to protein coding sequences representing 123,289 codons in 21,064 genes. The median (mean) percentage of missing data per SNP, including triallelic sites, was 1.06% (2.52%), whereas the percentage of heterozygous sites was 1.08% (2.52%). Only 4.5% of SNPs had more than 10% missing data (Figure S1A), and 0.18% had more than 10% heterozygous genotypes (Figure S1B).

We estimated error rates by first comparing our genotyped inbred B73 to the B73 reference genome, then by comparing all our genotypes to those from 7225 overlapping SNPs on the maize SNP50 bead chip (Cook *et al.* 2012). Compared with the reference genome, our B73 genotype differed (alternative homozygote allele) at 1.75% of SNPs, and across all lines our genotypes differed at a median (mean) rate of 1.83% (4.62%) from the maize SNP50 data (Cook *et al.* 2012).

### Statistical analyses

#### SNP annotation and analyses:

The first transcript of each gene in the B73 5b filtered gene set was used to annotate SNPs as synonymous and nonsynonymous with the software polydNdS from the analysis package of libsequence (Thornton 2003). The deleterious effects of amino acid changes were then predicted for proteins derived from the first transcript of each gene with both the SIFT (Ng and Henikoff, 2003, 2006) and MAPP (Stone and Sidow 2005) software packages.

SIFT uses homologous sequences identified by PSI-BLAST against protein databases to identify conserved amino acids. The software provides a scaled score of the putative deleterious effect of a particular amino acid at a position along a protein.

MAPP predicts deleterious amino acid polymorphisms from a user-defined alignment of protein homologs. It uses the phylogenetic relatedness among sequences and the physicochemical properties of amino acids to quantify the potential deleterious effect of a given amino acid change. We created alignments for MAPP using three different methods. First, we made BLASTX comparisons of protein sequences from maize against the TrEMBL database (Boeckmann *et al.* 2003) retaining all proteins with an e-value ≤ 10^−40^ and at least 60% identity with the query. Second, we used a reciprocal best BLAST criterion to compare protein sequences of maize against protein sequences from 31 plant genomes (File S2) from Phytozome version 8.0 (http://www.phytozome.net), retaining the best hit protein from each of the other genomes with an e-value ≤10^−100^ and ≥70% coverage of the query length. Finally, we made use of a set of syntenic genes from the grasses *Zea mays*, *Sorghum bicolor*, *Oryza sativa*, and *Brachypodium distachyon* (Schnable *et al.* 2012). For each set of proteins, ClustalW2 (Larkin *et al.* 2007) was used to align the sequences and build a neighbor-joining tree. Custom R code (https://github.com/RILAB/siftmappR) was used to link amino acid positions to SNP positions and to link the amino acid polymorphisms to MAPP and SIFT predictions.

The derived site frequency spectrum was calculated for all protein coding SNPs using *Tripsacum* (Chia *et al.* 2012) to determine ancestral state. The pattern of haplotype sharing (PHS) across the genome (PHS statistics; Toomajian *et al.* 2006) was analyzed within each of the tropical, stiff-stalk, non-stiff stalk, and mixed subpopulations as defined by Flint-Garcia *et al.* (2005). We will refer to these subpopulations as “genetic groups.”

#### Phenotypic data analyses:

Genetic values (the average phenotypic value of all individuals with the same genotype) of inbreds and hybrids in population B were taken from Flint-Garcia *et al.* (2009). Genetic values for population A were estimated from the raw phenotypic data using the model:

y=1μ+Xg+ε

where **y** is the vector of phenotypic values, *μ* is the mean of **y**, *X* is an incidence matrix, **g** is the vector of fixed individual effects, and *ε* comprises the residuals assumed to be $N\left(0,\sigma_{\varepsilon }^{2}I\right)$.

Hybrid vigor for each individual was estimated by both best- and mid-parent heterosis (*BPH* and *MPH*, respectively):

$$MPH_{ij}=\hat{g_{ij}}-\frac{1}{2}\left(\hat{g_{i}}+\hat{g_{j}}\right)$$

$$BPH_{min,ij}=\hat{g_{ij}}-min\left(\hat{g_{i}},\hat{g_{j}}\right)$$

$$BPH_{max,ij}=\hat{g_{ij}}-max\left(\hat{g_{i}},\hat{g_{j}}\right)$$

where $\hat{g_{ij}}$, $\hat{g_{i}}$, and $\hat{g_{j}}$ are the genetic values of the hybrid and its two parents *i* and *j*. *BPH_min_* was used instead of *BPH_max_* for days to anthesis, tassel branch count, tassel angle, and upper leaf angle.

#### Association mapping:

SNP association with the genetic values of the inbred lines was tested with the R package EMMA (Kang *et al.* 2008), following a mixed linear model similar to Yu *et al.* (2006):

$$\hat{g}=1\mu +M\vartheta +S\beta +Zu+\varepsilon$$

where $\hat{g}$ is the vector of estimated genetic values for inbred lines, *μ* is the mean of $\hat{g}$, *M* is the tested SNP, *ϑ* is the SNP effect, *S* indicates the structure covariates estimated by Flint-Garcia *et al.* (2005) using the STRUCTURE software (Pritchard *et al.* 2000), *β* indicates the fixed structure effects, *Z* is an incidence matrix, **u** is a vector of polygenic background effects assumed to be $N\left(0,\sigma_{u}^{2}K\right)$, and *ε* comprises the model residuals assumed to be $N\left(0,\sigma_{\varepsilon }^{2}I\right)$. The coancestry matrix *K* among inbred lines was approximated by an identity by state matrix calculated with the SNPs. Only SNPs with a minor allele frequency ≥0.05 were used for association mapping tests.

In hybrids, we tested the effect of heterozygosity at a given locus on observed heterosis. Each SNP was assigned numerical values corresponding to 0 if the hybrid is homozygous or 1 if the hybrid is heterozygous. The association mapping tests were thus carried out between heterozygosity at a given locus and hybrid vigor:

$$PH=1\mu^{\prime }+D\beta +H\vartheta +\varepsilon^{\prime }$$

where *PH* is the vector of heterosis values (either *MPH*, *BPH_max_*, or *BPH_min_*), *μ*′ is the mean of *PH*, *D* is the genetic distance between the tester (B73 or Mo17) and each inbred line, *β* is the fixed effect of that distance, *H* is the tested locus (1 if heterozygote and 0 if homozygote), *ϑ* the effect of the locus, and *ε*′ is the vector of residuals assumed to be $N\left(0,\sigma_{\varepsilon \prime }^{2}I\right)$. SNPs were deemed to be statistically significant at *P* ≤ 0.001. Analyses also were conducted in which we controlled for a false-discovery rate (Benjamini and Hochberg 1995) at 20%.

## Results and Discussion

### Prediction of deleterious mutations

To investigate deleterious mutations in a diverse set of maize inbred lines, we first applied two complementary approaches to predict deleterious mutations across the maize genome. We applied the software packages SIFT (Ng and Henikoff, 2003, 2006) and MAPP (Stone and Sidow 2005) to the 39,656 genes in version 5b of the maize filtered gene set (http://www.maizesequence.org; Schnable *et al.* 2009). SIFT predicted amino acid change consequences for nearly 12 million codons in 32,000 genes, whereas MAPP obtained predictions for a total of 11 million codons in 29,000 genes combined across the three ortholog datasets used (see the section *Materials and Methods*). More than 80% of predictions were congruent between the two approaches, similar to what has been seen in *Arabidopsis* and rice (Günther and Schmid 2010). SIFT and MAPP respectively identified ~80% and 60% of amino acid polymorphisms as “tolerated,” with the remainder predicted to be premature stop codons or “non-tolerated” amino acid changes; we will refer to these latter categories as predicted deleterious SNPs.

We then took advantage of recently published GBS (Elshire *et al.* 2011) data to survey potentially deleterious mutations across a panel of 247 diverse maize inbred lines (Larsson *et al.* 2013; Romay *et al.* 2013). The genotyping data include a total of 437,650 SNPs covering 123,289 codons. SIFT and MAPP predictions were obtained for 112,326 and 107,472 codons representing 19,145 and 18,255 genes, respectively (Figure S2). Nearly 50% of these codons showed no amino acid polymorphism in each dataset; although the vast majority of these monomorphic amino acids were attributable to synonymous polymorphisms in the GBS data, several hundred predicted deleterious amino acids were fixed across all maize lines analyzed (Table S2). Combining results from both SIFT and MAPP, our data consist of 25,352 predicted deleterious SNPs in 11,034 genes.

### Characterization of deleterious SNPs in a diversity panel

**Figure 1:**
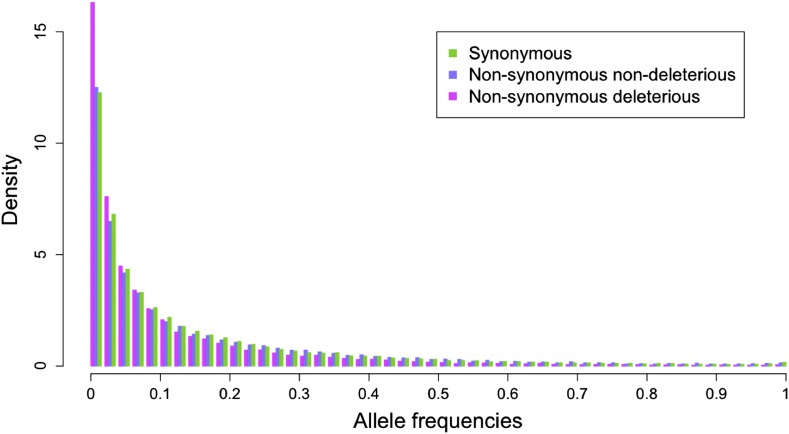
Derived site frequency spectrum of synonymous, nonsynonymous nondeleterious, and nonsynonymous deleterious SNPs. *Tripsacum* was used as outgroup for identifying the derived allele.

Across all lines, the derived site frequency spectrum (SFS) of coding SNPs showed an excess of rare variants compared with neutral expectations, with 45% of predicted deleterious SNPs occurring at derived frequencies less than 5% in the SFS across all lines. Even so, nonsynonymous SNPs showed an excess of rare variants compared with synonymous SNPs (Mann-Whitney *U*-test *P* < 10^−15^), and predicted deleterious SNPs showed a marked excess of rare variants compared with both synonymous and non-deleterious nonsynonymous variants (Mann-Whitney *U*-test *P* < 10^−15^ for both comparisons; Figure 1). The SFS of nondeleterious nonsynonymous was not distinguishable from that of synonymous variants (Mann-Whitney *U*-test *P* = 0.07). These observations are consistent with the action of weak purifying selection (Cummings and Clegg 1998; Fay *et al.* 2001) and independent corroboration of the utility of MAPP and SIFT in predicting deleterious variants.

Although most predicted deleterious alleles were rare, 923 were found segregating at high frequency (≥0.80) across all lines. To test whether these alleles may have been driven to high frequency by selection at linked loci during domestication (Lu *et al.* 2006), we analyzed the pattern of haplotype sharing across the genome (PHS statistics; Toomajian *et al.* 2006). Only 87 of these SNPs (9.4% of all tests) showed signs of positive selection in at least one of the genetic groups, and only 25 (2.7%) were found in candidate regions for selection during maize domestication (Hufford *et al.* 2012), providing little evidence to support hitchhiking during domestication as a major influence on the distribution of deleterious alleles in the genome.

**Figure 2:**
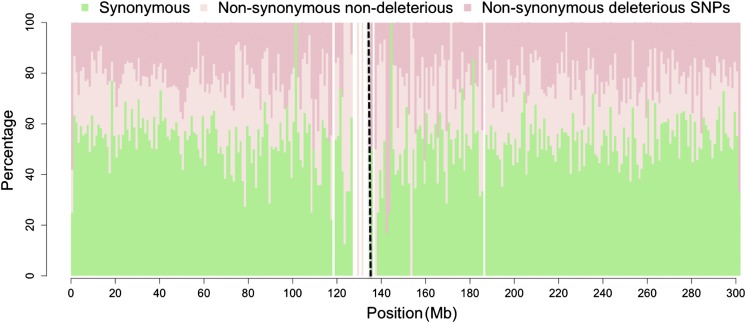
Proportion of genic SNPs predicted to be synonymous, nonsynonymous nondeleterious, and nonsynonymous deleterious in 1-Mb windows along chromosome 1. The vertical dashed black line indicates the centromere position, and blank columns indicate windows with missing data.

The proportion of genic SNPs predicted to be deleterious appeared relatively uniform (Figure 2 and Figure S3) across the genome, showing a very low correlation with recombination rate (Pearson’s *r* of 0.06; *P* = 0.005) from the IBM (Intermated B73xMo17) genetic map (Gerke *et al.* 2013). Explicit comparison of 1778 nonsynonymous pericentromeric (± 5 cM around the functional centromere) SNPs did not show an elevated proportion of predicted deleterious SNPs in comparison to the whole genome (Fisher’s exact test *P* = 0.68), and no correlation was observed between gene density and the proportion of predicted deleterious mutations in 1 Megabase windows (Pearson’s *r* of −0.06; *P* = 0.01). The negative correlation between recombination and residual heterozygosity observed in recombinant inbred lines of the maize nested association mapping population has been attributed to the inefficiency of selection against deleterious alleles in low recombination regions of the genome (McMullen *et al.* 2009; Gore *et al.* 2009). Our results do not provide support for this explanation, perhaps suggesting that recombination in these regions over longer periods of time is sufficient to avoid the accumulation of deleterious alleles. Consistent with this idea, although regions of the *Drosophila* genome completely lacking in recombination showed a severe reduction in the efficacy of selection, little difference was observed between regions with high and low rates of recombination (Haddrill *et al.* 2007).

Individual lines varied considerably in their content of predicted deleterious alleles, carrying between 4 and 16% of all predicted deleterious alleles. Lines from the stiff-stalk group carried on average fewer deleterious mutations (9%) than did lines from other groups (14–15%), even after weighting by the total SNPs in each group (data not shown). Although drift due to a historically low *N_e_* (Messmer *et al.* 1991) could explain this observation, other groups with low *N_e_* such as the popcorns do not show such a trend. Instead, we posit that both the SIFT and MAPP algorithms may be biased against identifying deleterious alleles found in the reference B73 genome. Because B73 is a stiff-stalk line and both programs use the reference allele in identifying deleterious alleles, nonsynonmyous SNPs at appreciable frequency in the stiff-stalk group may be more likely falsely identified as tolerated. Similar bias has recently been described in analyses of the human genome (Simons *et al.* 2013).

Allele sharing at predicted deleterious SNPs generally followed genome-wide patterns of identity by state (IBS). Within the non-stiff stalk, tropical, popcorn, and sweet groups, correlations were generally high (Pearson’s *r* of 0.75−0.99) between numbers of shared predicted deleterious alleles (mean of 5−10%) and IBS. Correlations between inbreds from different genetic groups were much lower (*r* of 0.25−0.52), however, as has been previously seen in correlations between IBS and heterosis observed at SSR loci (Flint-Garcia *et al.* 2009). The “mixed” (within group *r* = 0.22 and *r* = −0.05 to 0.36 with other groups) and stiff-stalk (within-group *r* = 0.15 and *r* = −0.65 to 0.16 with other groups) groups appeared exceptions to this pattern, perhaps because of the aforementioned ascertainment bias or previously unrecognized population substructure within these groups (Figure S4).

**Figure 3:**
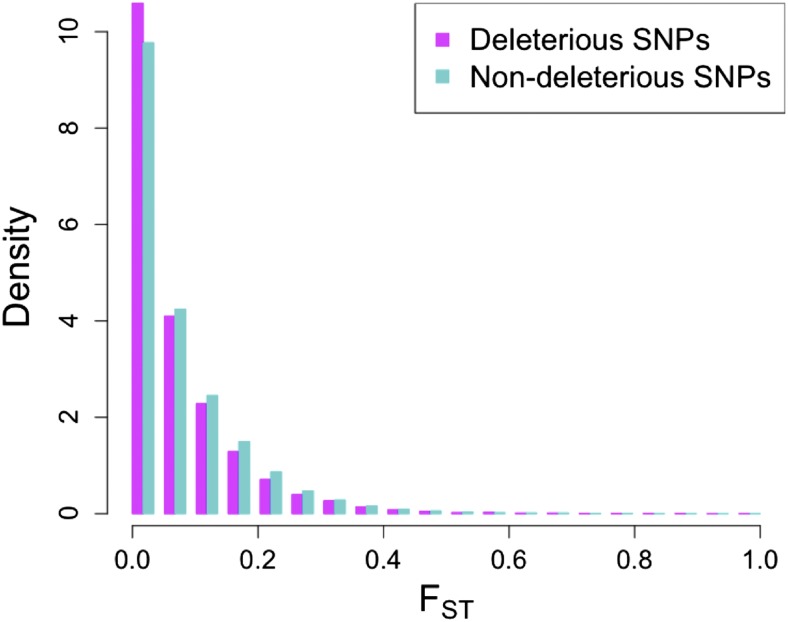
F_ST_ distribution for deleterious and nondeleterious SNPs.

**Figure 4:**
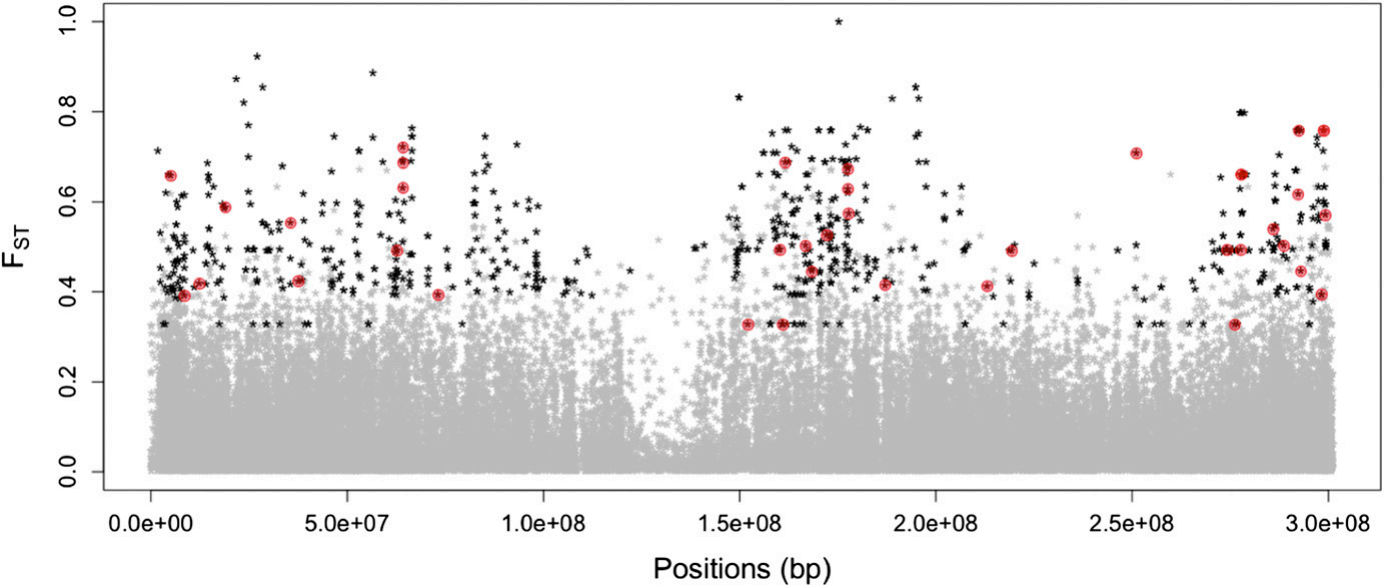
Distribution of F_ST_ along chromosome 1. Black dots represent SNPs in the top 1% of F_ST_ and those predicted to be deleterious are surrounded in red.

Across all genetic groups, levels of population differentiation were slightly lower for predicted deleterious (mean F_ST_ = 0.07) than nondeleterious (mean F_ST_ = 0.08) SNPs (Mann-Whitney *U*-test *P* < 10^−15^; Figure 3). After correcting for allele frequencies in both classes, however, these differences disappeared and the proportion of deleterious SNPs in the top 1% of F_ST_ was not significantly different from the proportion observed for synonymous SNPs (Fisher’s exact test *P* = 0.94) or all SNPs in genic regions (Fisher’s exact test *P* = 0.51). After allele frequency correction, 287 genes had a predicted deleterious SNP in the top 1% of Fst among genetic groups, and 30 genes had two or more high-Fst predicted deleterious SNPs (see Figure 4 for chromosome 1). Only 11 genes (4%) with high-Fst deleterious SNPs are found in regions thought to be selected during maize improvement (Hufford *et al.* 2012) and only 44 of the 287 genes (15%) show significant signs of positive selection with the PHS statistic. Neither result provides much evidence that selection on linked beneficial mutations strongly impacts frequencies of deleterious alleles.

**Figure 5:**
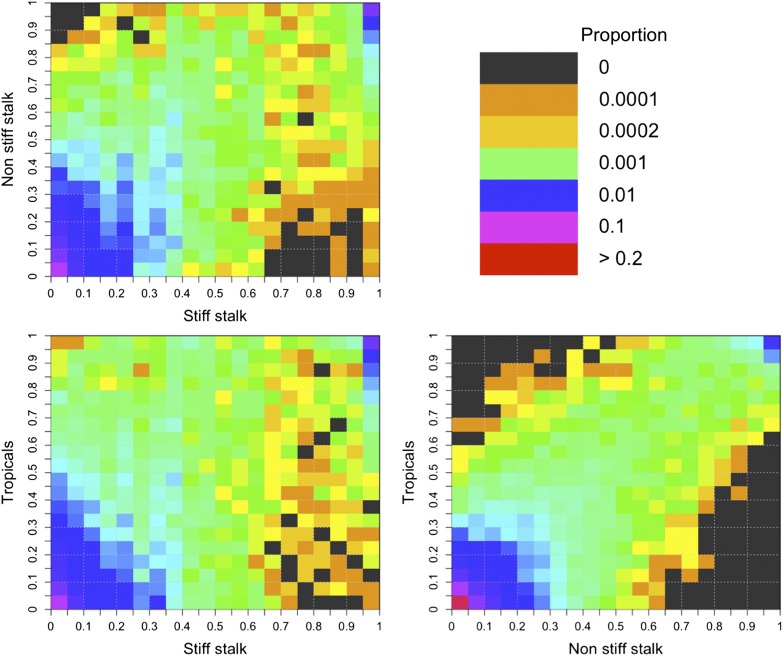
Joint site frequency spectrum of stiff-stalk, non-stiff stalk, and tropical genetic groups. Axes represent the frequency of the predicted deleterious alleles in a group and colors show the proportion of SNPs at a given frequency.

Comparisons of the predicted deleterious SFS between stiff-stalk, non-stiff stalk, and tropical groups (Figure 5) mirrored patterns of between-group F_ST_, revealing few fixed differences between groups and generally low frequencies within groups, as well as higher differentiation in comparisons involving the stiff-stalk group.

Observed frequencies of deleterious SNPs in different populations (Figure 5) may help explain patterns of hybrid vigor. Although F_ST_ is generally low, inbreds from different genetic groups are nonetheless likely to share fewer deleterious variants than inbreds from the same group, and heterosis is higher among crosses between groups (Figure S5). Nonetheless, even crosses among inbreds from the same genetic group show evidence of heterosis (Figure S5), likely due to the large number of deleterious SNPs segregating at low frequencies within individual populations.

### Effect of deleterious mutations on traits of interest

To investigate the contribution of predicted deleterious alleles to observed levels of heterosis and inbreeding depression, we performed a genome-wide association analysis of 17 traits evaluated in two populations (see the section *Materials and Methods*). Analyses were carried out using the genetic values of inbred lines and both mid-parent and best-parent heterosis. Genome-wide association results using the genetic values of inbred lines identified between 219 (cob diameter) and 598 (cob length) significant SNPs with a high proportion of genic loci (up to 70%) but little evidence for significant enrichment of predicted deleterious SNPs (Table 1 and Table S4).

**Table 1:** Total number of significant SNPs in genic regions (*n*) and fold enrichment (*f*) for deleterious SNPs in population A

	Inbreds		BPH		MPH	
Traits	*n*	*f*	*n*	*f*	*n*	*f*
Days to tasseling	475	1.05	3372	1.15*^a^*	1123	1.12
Tassel length	458	0.81	297	1.21	365	1.16
Tassel branch count	300	0.98	4077	0.98	1257	1.12
Tassel angle	244	1.11	490	0.93	646	1.18
Plant height	282	0.92	18068	0.98	9712	0.93
Upper leaf angle	415	1.20	8927	0.99	2266	1.12
Leaf width	289	1.21	1064	1.16	1051	1.01
Leaf length	389	1.14	4256	0.93	2257	1.07
Kernel height	292	1.10	8752	1.08	4512	1.01
Stem puncture resistance	258	0.79	443	1.04	375	0.93
Plant yield	257	1.50	7440	1.12*^a^*	7007	1.14*^a^*
Ear length	231	0.89	605	1.11*^a^*	907	1.00
10 kernel weight	298	1.29	709	1.15	761	1.30
Cob diameter	219	1.04	4363	1.16*^a^*	405	0.88
Cob weight	228	1.09	1746	0.93	519	0.69
Kernel weight	256	0.88	3781	0.98	2045	0.95

SNP, single-nucleotide polymorphism; BPH, best-parent heterosis; MPH, mid-parent heterosis.

a Statistically significant (Fisher’s exact test *P* < 0.05).

Results for association between SNP heterozygosity and heterosis showed highly variable numbers of significant loci (Table 1 and Table S4), also with a high proportion of genic SNPs (up to 74%). Significant loci explained between 4 and 40% of the observed phenotypic variation in heterosis, although these values are likely inflated due to small sample size (Beavis 1994). The greatest numbers of associated SNPs were observed for plant height and yield-related traits, which also showed the greatest levels of observed heterosis. Furthermore, most traits exhibited some enrichment (5−45%) of predicted deleterious SNPs and the enrichment was statistically significant for whole plant yield and days to tasseling. These enrichment results hold even after an FDR control at 20% and similar enrichments were observed when comparing nonsynonymous deleterious with nonsynonymous nondeleterious SNPs (data not shown). Although crosses to both Mo17 and B73 showed evidence of enrichment in population B, only crosses to B73 were statistically significant, likely due to the lower number of deleterious SNPs identified in the stiff-stalk heterotic group.

Because most deleterious SNPs are at frequencies too low for inclusion in association analyses (Figure 1), we expanded our test of enrichment to the gene level, asking whether genes with predicted deleterious SNPs were more likely than random to have SNPs significantly associated with traits of interest. At this level we see much stronger evidence of enrichment, even with an FDR control at 20%: a number of traits show statistically significant enrichment in population A, but virtually all traits in both populations show a positive enrichment for genes with predicted deleterious SNPs (Table 2 and Table S5), a result that is highly unlikely by chance (sign test *P* = 3 × 10^−5^ for population A and 0.01 for population B). Similar tests of low-frequency synonymous SNPs show no evidence of enrichment (*P* ≈ 1), and the low correlation between total SNPs in a gene and the number of significant associations (*r* ≤ 0.2) suggests that our observation is not an artifact of the number of SNPs analyzed per gene. Furthermore, the enrichment result holds for groups of genes with similar numbers of SNPs.

**Table 2:** Total number of genes with significant SNPs (*n*) and fold enrichment for genes with predicted deleterious SNPs (*f*) in population A

	Inbreds		BPH		MPH	
Traits	*n*	*f*	*n*	*f*	*n*	*f*
Days to tasseling	176	1.11	1137	1.12*^a^*	429	1.15*^a^*
Tassel length	173	1.08	128	1.14	154	1.20
Tassel branch count	114	1.02	1257	1.13*^a^*	472	1.14*^a^*
Tassel angle	103	1.03	177	1.10	254	1.15
Plant height	128	1.22	4529	1.10*^a^*	2741	1.10*^a^*
Upper leaf angle	166	1.13	2553	1.11*^a^*	810	1.15*^a^*
Leaf width	112	1.27	379	1.05	375	1.14
Leaf length	141	1.18	1290	1.13*^a^*	821	1.20*^a^*
Kernel height	123	1.09	2633	1.13*^a^*	1506	1.14
Stem puncture resistance	99	1.24	164	1.10	145	1.07
Plant yield	117	1.22	2440	1.14*^a^*	2302	1.14*^a^*
Ear length	84	1.02	230	1.20	333	1.15
10 kernel weight	137	1.18	288	1.17	308	1.13
Cob diameter	90	1.10	1419	1.13*^a^*	162	1.12
Cob weight	99	1.19	548	1.07	176	1.13
Kernel weight	101	1.18	1228	1.11*^a^*	714	1.07

SNP, single-nucleotide polymorphism; BPH, best-parent heterosis; MPH, mid-parent heterosis.

a Statistically significant (Fisher’s exact test P < 0.05).

We posit that the observed excess of significant associations in genes with predicted deleterious variants may be due to so-called synthetic associations between rare deleterious alleles and a common allele at a linked locus at high enough frequency to be included in association mapping tests (Goldstein 2009; Dickson *et al.* 2010). Recent work suggests that this sort of association is only likely to hold for deleterious alleles with a relatively small effect on phenotype (Thornton *et al.* 2013), which is consistent with the expected weak-to-intermediate effects of deleterious alleles likely to be involved in heterosis (Charlesworth and Charlesworth 1987; Whitlock *et al.* 2000; Glémin *et al.* 2003; Charlesworth and Willis 2009). Strongly deleterious alleles, although potentially playing a role in inbreeding depression (Whitlock *et al.* 2000), are less likely to be observed in our study as selection should effectively remove them from our panel of inbred lines.

Although we have analyzed only a relatively small subset of the genome-wide diversity of maize (Chia *et al.* 2012), our data nonetheless present the first genome-wide scan of deleterious coding variants in maize. Our results provide evidence for the contribution of deleterious mutations to heterosis via complementation, consistent with the dominance hypothesis. The weak expected effects of these deleterious SNPs, combined with their low frequencies, make their detection difficult using conventional approaches. *A priori* prediction of the potential effect of rare polymorphisms, however, may improve predictions of inbred line breeding values and combining ability. Future analysis of full genome sequence data, allowing for the inclusion of all coding SNPs and noncoding variants, will provide an even richer catalog of variants that will expand our understanding of the role of rare deleterious variants in maize breeding.

## Supplementary Material

Supporting Information
