# Supplementary material for: The Pattern and Distribution of Deleterious Mutations in Maize
Source: G3 (Bethesda). 2013 Nov 26;4(1):163–71. doi: 10.1534/g3.113.008870 (PMC3887532; doi:10.1534/g3.113.008870)
Supplement: Supporting Information [file supp_4_1_163__index.html]

The Pattern and Distribution of Deleterious Mutations in Maize — Supporting Information 

# The Pattern and Distribution of Deleterious Mutations in Maize

## Supporting Information for Mezmouk and Ross-Ibarra, 2014

**Files in this Data Supplement:**

- Supporting Information - Figures S1-S5, Files S1-S4, and Tables S1-S5 (PDF, 1 MB)
- Figure S1 - Histograms of the percentage of (A) heterozygosity and (B) missing data per SNP. (PDF, 135 KB)
- Figure S2 - Comparison of the number of predicted (A) amino acids and (B) genes, covered by SNP data. (PDF, 351 KB)
- Figure S3 - Proportion of genic SNPs predicted to be synonymous, non-synonymous non-deleterious and non-synonymous deleterious in 1 cM windows along chromosome 1. (PDF, 547 KB)
- Figure S4 - Projection of the (A) stiff stalk and (B) mixed inbred lines on the three first axes of a principal component analysis. (PDF, 163 KB)
- Figure S5 - Distribution of best parent heterosis (BPH) for plant yield in population A. (PDF, 139 KB)
- File S1 - List of the inbred lines used. (PDF, 54 KB)
- File S2 - List of genomes used for reciprocal BLAST. (PDF, 59 KB)
- Table S1 - List of Analyzed traits (PDF, 44 KB)
- Table S2 - Detailed results of the prediction of deleterious amino acids with MAPP, using the different gene sets, and with SIFT. (PDF, 44 KB)
- Table S3 - Comparison of the results of MAPP predictions with the different gene sets. (PDF, 43 KB)
- Table S4 - Total number of significant SNPs in genic regions (*n*) and fold enrichment (*f*) for SNPs with deleterious mutations in population B. (PDF, 75 KB)
- Table S5 - Total number of genes with significant SNPs (*n*) and fold enrichment for genes with predicted deleterious SNPs (*f*) in population B. (PDF, 75 KB)
- File S3 - SNP data (.zip, 23 MB)
- File S4 - Phenotypic data (.zip, 412 KB)
