# Supplementary material for: The Pattern and Distribution of Deleterious Mutations in Maize
Source: G3 (Bethesda). 2013 Nov 26;4(1):163–71. doi: 10.1534/g3.113.008870 (PMC3887532; doi:10.1534/g3.113.008870)
Supplement: Supporting Information [file supp_g3.113.008870_008870SI.pdf]

G3· Genes | Genomes | Genetics

The Pattern and Distribution of Deleterious Mutations in Maize

Sofiane Mezmouk[*1] and Jeffrey Ross-Ibarra[†1,2]

[1]Department of Plant Sciences, University of California Davis
[2]Center for Population Biology and Genome Center, University of California Davis

[*]smezmouk@ucdavis.edu
[†]rossibarra@ucdavis.edu

DOI: 10.1534/g3.113.008870

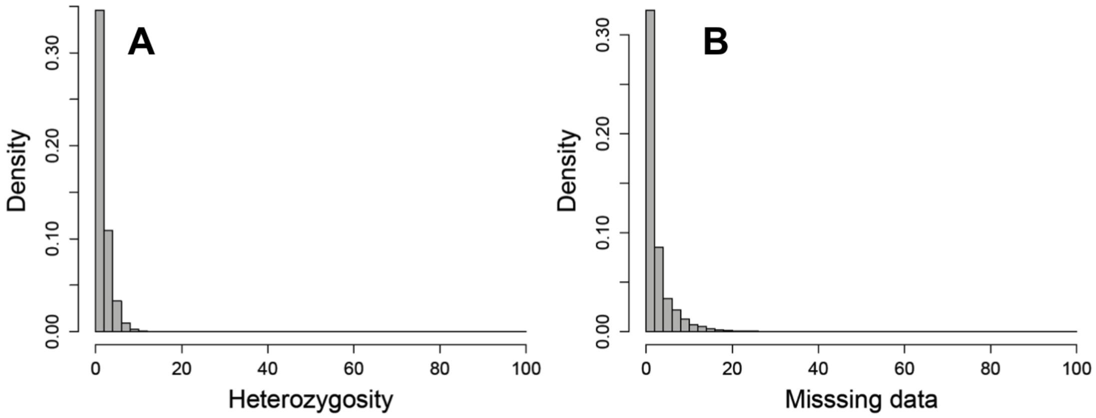

Figure S 1: Histograms of the percentage of (A) heterozygosity and (B) missing data per SNP

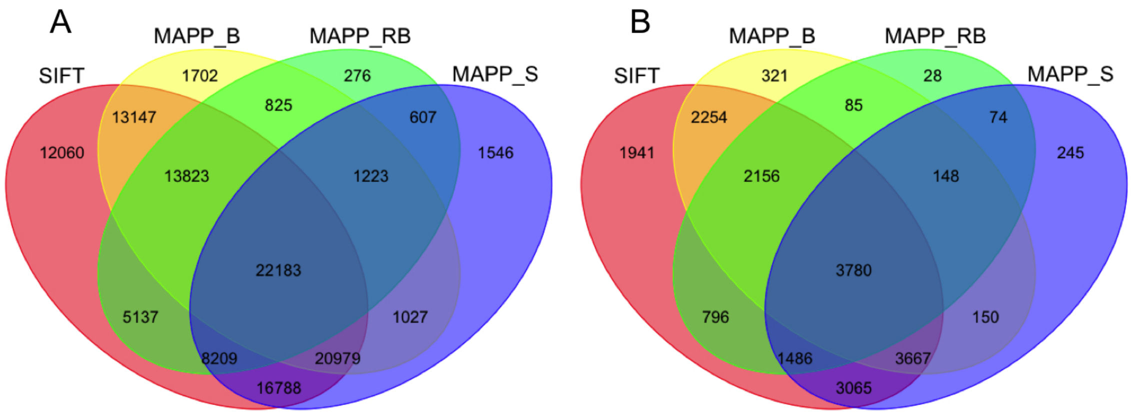

Figure S 2: Comparison of the number of predicted (A) amino acids and (B) genes, covered by SNP data. For MAPP, 3 gene sets were used: BLASTX (MAPP_B), reciprocal BLAST (MAPP_RB) and syntenic genes (MAPP_S)

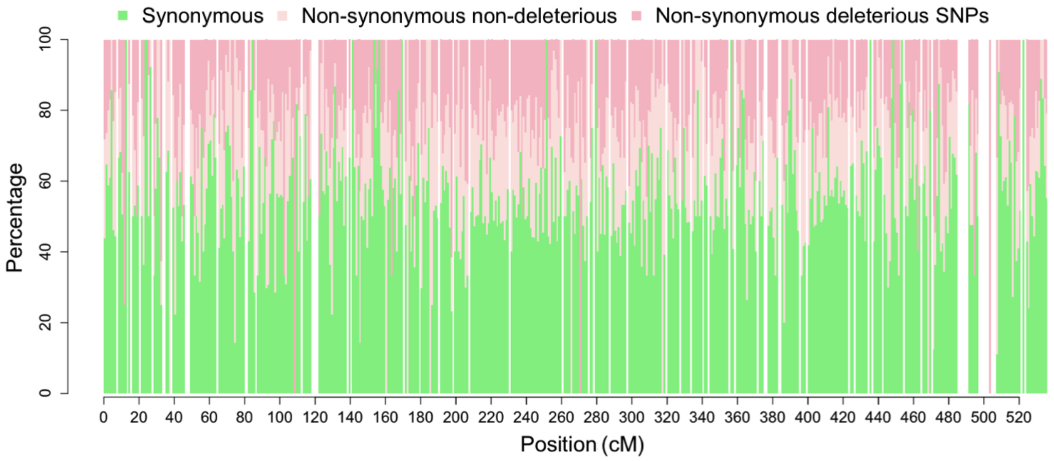

Figure S 3: Proportion of genic SNPs predicted to be synonymous, non-synonymous non-deleterious and non-synonymous deleterious in 1 cM windows along chromosome 1

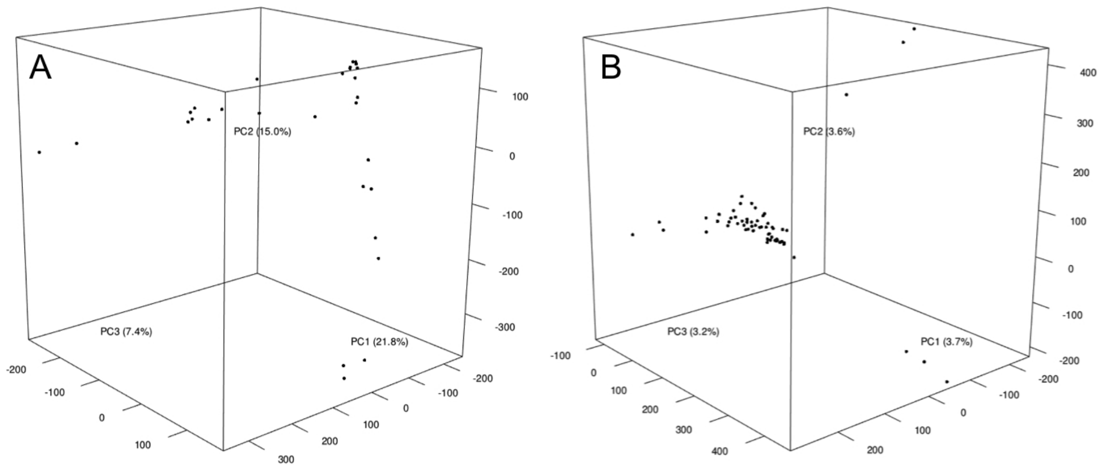

Figure S 4: Projection of the (A) stiff stalk and (B) mixed inbred lines on the three first axes of a principal component analysis

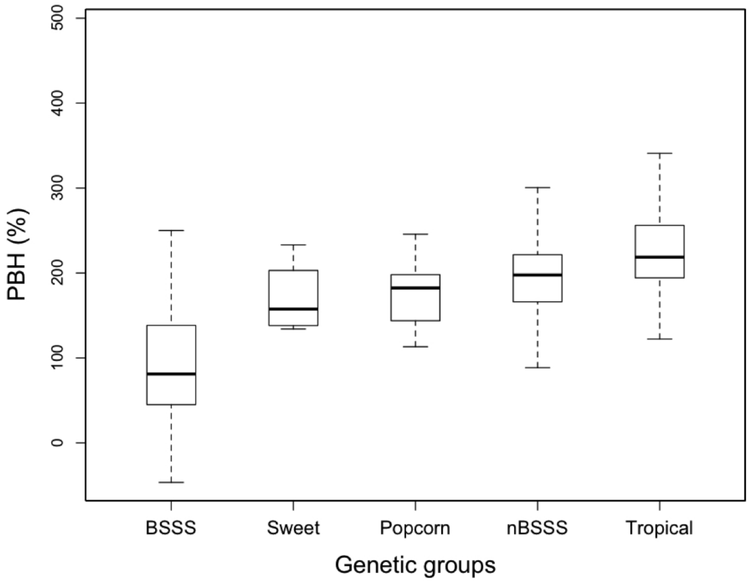

Figure S 5: Distribution of best parent heterosis (BPH) for plant yield in population A. BSSS and nBSSS indicate the stiff stalk and non-stiff stalk groups.

File S1

List of the inbred lines used

PopulationA

B73, A214N, A441.5, A554, A556, A6, A619, A632, A634, A635, A641, A654, A659, A661, A679, A680, A682, AB28A, B10, B104, B105, B109, B115, B14A, B164, B2, B37, B46, B57, B64, B68, B73HTRHM, B75, B76, B77, B79, B84, B97, CH701.30, CH9, CI187.2, CI21E, CI28A, CI31A, CI3A, CI64, CI66, CI7, CI90C, CI91B, CM174, CM37, CM7, CML10, CML103, CML108, CML11, CML14, CML154Q, CML157Q, CML158Q, CML218, CML220, CML228, CML238, CML247, CML258, CML261, CML264, CML277, CML281, CML287, CML311, CML314, CML321, CML322, CML323, CML328, CML331, CML332, CML333, CML341, CML38, CML5, CML52, CML69, CML77, CML91, CML92, CMV3, CO255, D940Y, DE1, DE2, DE811, E2558W, EP1, F2834T, F44, F6, GA209, GT112, H105W, H84, H91, H95, H99, HI27, HP301, HY, I137TN, I205, I29, IA2132, IA5125, IDS28, IDS69, IDS91, IL101T, IL14H, IL677A, K148, K4, K55, K64, KI11, KI14, KI2021, KI21, KI3, KI43, KI44, KY21, KY226, KY228, L317, L578, M14, M162W, M37W, MEF156.55.2, MO17, MO18W, MO1W, MO24W, MO44, MO45, MO46, MOG, MP339, MS1334, MS153, MS71, MT42, N192, N28HT, N6, N7A, NC222, NC230, NC232, NC236, NC238, NC250, NC258, NC260, NC262, NC264, NC294, NC296, NC296A, NC298, NC300, NC302, NC304, NC306, NC310, NC314, NC318, NC320, NC324, NC326, NC328, NC33, NC336, NC338, NC342, NC344, NC346, NC348, NC350, NC352, NC354, NC356, NC358, NC360, NC362, NC364, NC366, NC368, ND246, OH40B, OH43E, OH603, OH7B, OS420, P39, PA762, PA875, PA880, PA91, R168, R177, R229, R4, SA24, SC357, SC55, SD44, SG1533, SG18, T232, T8, TX303, TZI10, TZI11, TZI16, TZI18, TZI25, TZI8, TZI9, U267Y, VA102, VA14, VA22, VA35, VA59, VA99, VAW6, W117HT, W153R, W182B, W64A, WD, 33.16, 38.11, X4226, X4722

PopulationB

B73, MO17, 33.16, A188, A239, A619, A632, A634, A635, A641, A654, A661, A679, A680, A682, B103, B104, B109, B115, B14A, B37, B46, B52, B57, B64, B68, B73, B73HTRHM, B75, B76, B77, B79, B84, C103, C49A, CH701.30, CM105, CM174, CO125, DE.2, DE1, DE811, EP1, H105W, H49, H84, H91, H95, H99, HP301, IL101, IL14H, K148, KY226, M14, MEF156.55.2, MO44, MO45, MO46, MO47, MS1334, MS153, MS71, N192, N28HT, N6, NC262, NC264, NC294, NC306, NC310, NC314, NC324, NC326, NC328, NC342, NC364, ND246, OH43, OH43E,

OS420, P39, PA762, PA875, PA880, PA91, R168, R177, R4, SD40, SD44, SG18, VA102, VA14, VA17, VA22, VA35, VA85, VA99, W182B, W22, W64A, WF9, YU796.NS.

File S2

List of genomes used for reciprocal BLAST

Aquilegia coerulea, Arabidopsis lyrata, Arabidopsis thaliana, Brachypodium distachyon, Brassica rapa, Capsella rubella, Carica papaya, Chlamydomonas reinhardtii, Citrus clementina, Citrus sinensis, Cucumis sativus, Eucalyptus grandis, Glycine max, Linum usitatissimum, Malus domestica, Manihot esculenta, Medicago truncatula, Mimulus guttatus, Oryza sativa, Panicum virgatum, Phaseolus vulgaris, Physcomitrella patens, Populus trichocarpa, Prunus persica, Ricinus communis, Selaginella moellendorffii, Setaria italica, Sorghum bicolor, Thellunigiella halophila, Vitis vinifera, Volvox carteri.

File S3-4

Available for download as .zip files at http://www.g3journal.org/lookup/suppl/doi:10.1534/g3.113.008870/-/DC1

File S3 *Phenotypic data*

File S4 *SNP data*

Table S 1: List of Analyzed traits

Traits	Abbreviation	Populations
Days to tasseling	DTT	A
Tassel length (cm)	TSLLEN	A
Tassel branch count	TSLBCHCNT	A
Tassel angle	TSANG	A
Plant height (cm)	PLTHT	A & B
Upper leaf angle	UPLFANG	A
Leaf width (cm)	LFWDT	A
Leaf length (cm)	LFLEN	A
Kernel height	KNLHGT	A
Kernel weight	TOTKNLWT	A
Stem puncture resistance (kg/section)	RPR	A
Plant yield (g/plant)	PLTYLD	A
Ear length (cm)	EARLGH	A & B
10 kernel weight (g)	10KWT	A
Cob diameter (cm)	COBDIA	A & B
Cob weight (g)	COBWT	A & B
Seed number per ear	SEEDNB	B

Table S 2: Detailed results of the prediction of deleterious amino acids with MAPP, using the different gene sets, and with SIFT

	MAPP			SIFT
Gene sets	BLASTX	Reciprocal BLAST	Syntenic genes	PSI-BLAST
Total a.a. positions with predictions	7,746,638	5,570,035	6,869,010	11,906,167
Total number of genes	20,348	11,918	17,957	31,843
Number of positions covered by SNPs	74,909	52,283	72,562	112,326
Number of genes covered by SNPs	12,561	8,553	12,615	19,145
Monomorphic tolerated	39,009	25,270	39,300	58,685
Monomorphic not tolerated*	144	3470	14	387
Polymorphic tolerated	18,379	10,753	17,792	42,606
Polymorphic not tolerated*	17,377	12,790	15456	10,648

*Includes premature stop codons

Table S 3: Comparion of the results of MAPP predictions with the different gene sets.

Gene sets	BLASTX	Reciprocal BLAST	Syntenic genes
BLASTX	-	80.1%	78.2%
Reciprocal BLAST	38,054 (6,169)	-	79.8%
Syntenic genes	45,412 (7,745)	32,222 (5,488)	-

The lower triangle indicates the number of amino acid positions predicted with two given gene sets and covered by GBS SNPs (number of genes between brackets); the upper triangle indicates the percentage of amino acids with the same predictions.

Table S 4: Total number of significant SNPs in genic regions (n) and fold enrichment (f) for SNPs with deleterious mutations in population B. Numbers marked with "*" are statistically significant (Fisher's exact test p-value<0.05).

	Inbreds		BPH_B73		MPH_B73		BPH_Mo17		MPH_Mo17	
Traits	*n*	*f*	*n*	*f*	*n*	*f*	*n*	*f*	*n*	*f*
10KWT	310	0.77	404	1.17*	257	0.86	698	0.83	723	0.98
COBWT	313	0.62	941	1.15*	387	0.69	257	1.33	532	0.95
COBDIA	226	1.49	159	1.25*	236	1.06*	349	0.78	615	0.72
COBLEN	598	1.08	239	1.20*	97	0.24	280	1.08	140	0.92
SEEDWT	362	1.09	378	1.32*	118	1.23*	1043	0.92	1080	0.78
SEEDNB	373	0.99	320	0.86	251	0.92	348	1.06	454	0.82
PLTHT	505	1.02	261	0.89	143	1.45*	1022	1.08	156	1.16

Table S 5: Total number of genes with significant SNPs (n) and fold enrichment for genes with predicted deleterious SNPs (f) in population B. Numbers marked with "*" are statistically significant (Fisher's exact test p-value$<$ 0.05).

	Inbreds		BPH_B73		MPH_B73		BPH_Mo17		MPH_Mo17	
Traits	n	f	n	f	n	f	n	f	n	f
10KWT	73	1.17	169	1.14	95	1.11	246	1.11	274	1.11
COBWT	71	1.13	316	1.08	128	1.04	94	1.10	204	1.10
COBDIA	81	1.07	57	1.08	86	1.11	134	1.03	234	1.14
COBLEN	203	1.09	89	1.24	30	1.17	110	1.17	51	1.21
SEEDWT	138	1.10	146	1.14	50	0.97	371	1.09	389	1.09
SEEDNB	106	1.15	128	1.13	116	0.98	130	1.12	166	1.09
PLTHT	169	1.15	112	1.09	65	1.13	348	1.15	65	1.15
