# Supplementary figures and images for: The Pattern and Distribution of Deleterious Mutations in Maize
Source: G3 (Bethesda). 2013 Nov 26;4(1):163–71. doi: 10.1534/g3.113.008870 (PMC3887532; doi:10.1534/g3.113.008870)

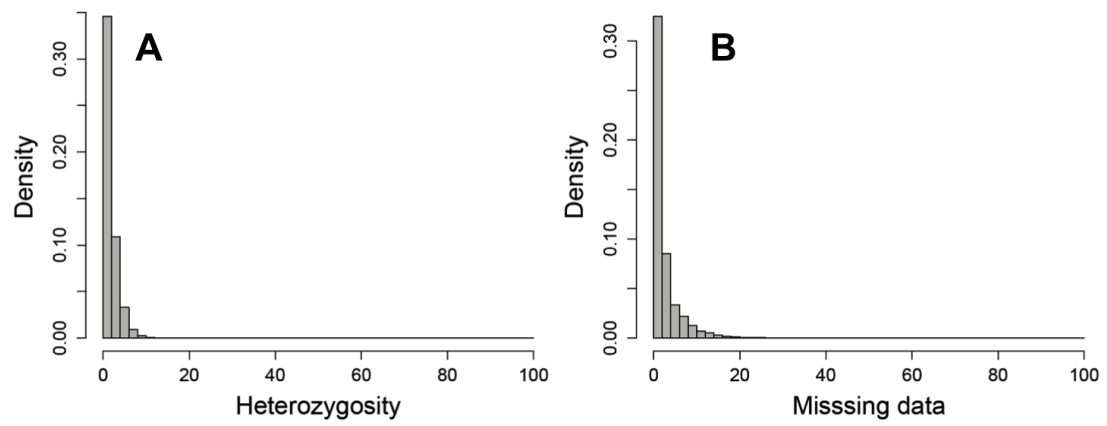

Figure S 1: Histograms of the percentage of (A) heterozygosity and (B) missing data per SNP

Supplement: Supporting Information [file supp_g3.113.008870_FigureS1.pdf]
