# Supplementary material for: The Pattern and Distribution of Deleterious Mutations in Maize
Source: G3 (Bethesda). 2013 Nov 26;4(1):163–71. doi: 10.1534/g3.113.008870 (PMC3887532; doi:10.1534/g3.113.008870)
Supplement: Supporting Information [file supp_g3.113.008870_FigureS2.pdf]

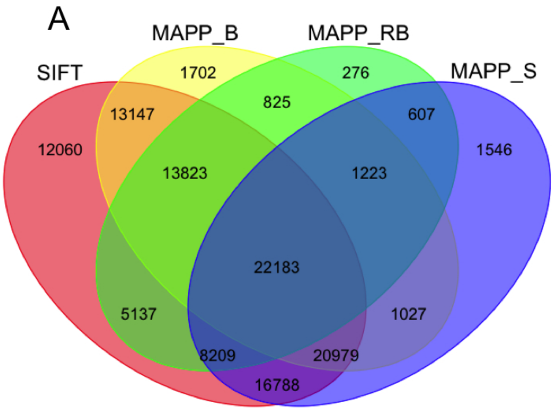

Figure S 2: Comparison of the number of predicted (A) amino acids and (B) genes, covered by SNP data. For MAPP, 3 gene sets were used: BLASTX (MAPP_B), reciprocal BLAST (MAPP_RB) and syntenic genes (MAPP_S)
