# Supplementary material for: The Pattern and Distribution of Deleterious Mutations in Maize
Source: G3 (Bethesda). 2013 Nov 26;4(1):163–71. doi: 10.1534/g3.113.008870 (PMC3887532; doi:10.1534/g3.113.008870)
Supplement: Supporting Information [file supp_g3.113.008870_FigureS3.pdf]

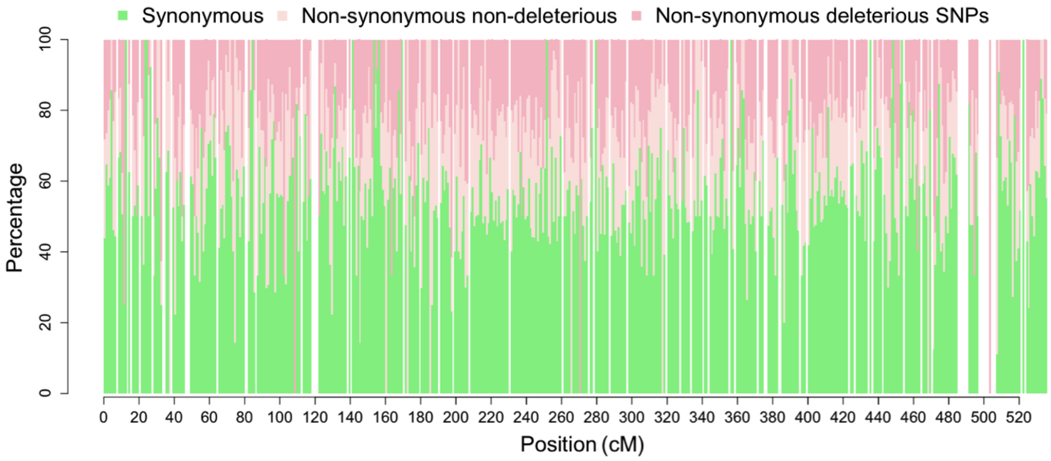

Figure S 3: Proportion of genic SNPs predicted to be synonymous, non-synonymous non-deleterious and non-synonymous deleterious in 1 cM windows along chromosome 1
