# Supplementary material for: The Pattern and Distribution of Deleterious Mutations in Maize
Source: G3 (Bethesda). 2013 Nov 26;4(1):163–71. doi: 10.1534/g3.113.008870 (PMC3887532; doi:10.1534/g3.113.008870)
Supplement: Supporting Information [file supp_g3.113.008870_FigureS4.pdf]

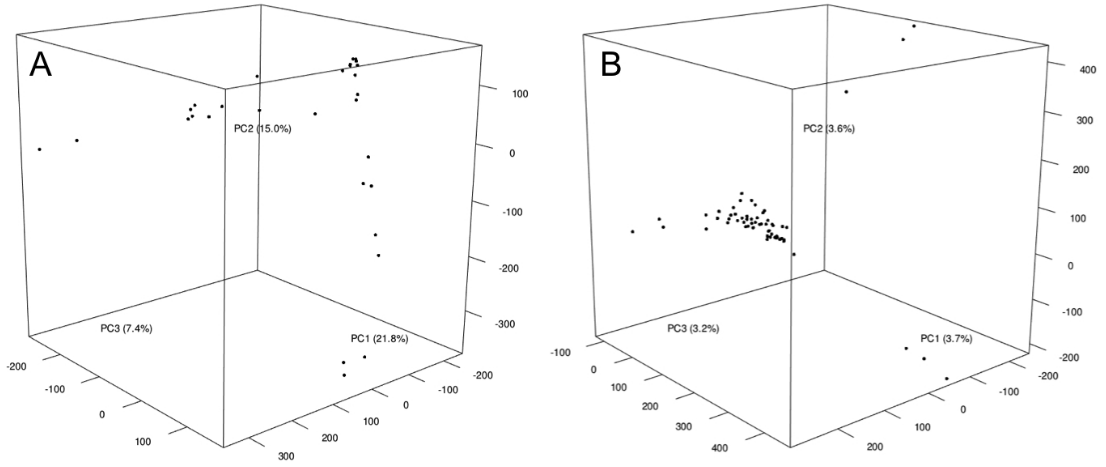


Figure S 4: Projection of the (A) stiff stalk and (B) mixed inbred lines on the three first axes of a principal component analysis
