# Supplementary material for: The Pattern and Distribution of Deleterious Mutations in Maize
Source: G3 (Bethesda). 2013 Nov 26;4(1):163–71. doi: 10.1534/g3.113.008870 (PMC3887532; doi:10.1534/g3.113.008870)
Supplement: Supporting Information [file supp_g3.113.008870_FigureS5.pdf]

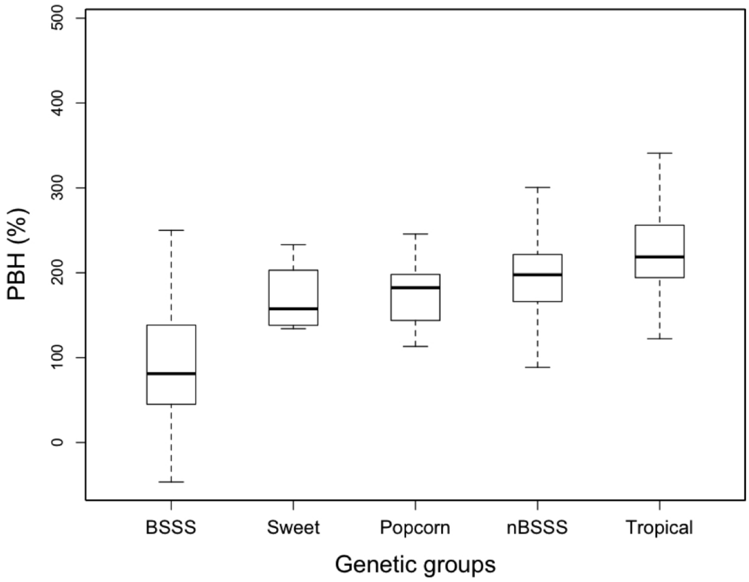

Figure S 5: Distribution of best parent heterosis (BPH) for plant yield in population A. BSSS and nBSSS indicate the stiff stalk and non-stiff stalk groups.
