# Supplementary material for: The Pattern and Distribution of Deleterious Mutations in Maize
Source: G3 (Bethesda). 2013 Nov 26;4(1):163–71. doi: 10.1534/g3.113.008870 (PMC3887532; doi:10.1534/g3.113.008870)
Supplement: Supporting Information [file supp_g3.113.008870_FileS1.pdf]

File S1

List of the inbred lines used

PopulationA

B73, A214N, A441.5, A554, A556, A6, A619, A632, A634, A635, A641, A654, A659, A661, A679, A680, A682, AB28A, B10, B104, B105, B109, B115, B14A, B164, B2, B37, B46, B57, B64, B68, B73HTRHM, B75, B76, B77, B79, B84, B97, CH701.30, CH9, CI187.2, CI21E, CI28A, CI31A, CI3A, CI64, CI66, CI7, CI90C, CI91B, CM174, CM37, CM7, CML10, CML103, CML108, CML11, CML14, CML154Q, CML157Q, CML158Q, CML218, CML220, CML228, CML238, CML247, CML258, CML261, CML264, CML277, CML281, CML287, CML311, CML314, CML321, CML322, CML323, CML328, CML331, CML332, CML333, CML341, CML38, CML5, CML52, CML69, CML77, CML91, CML92, CMV3, CO255, D940Y, DE1, DE2, DE811, E2558W, EP1, F2834T, F44, F6, GA209, GT112, H105W, H84, H91, H95, H99, HI27, HP301, HY, I137TN, I205, I29, IA2132, IA5125, IDS28, IDS69, IDS91, IL101T, IL14H, IL677A, K148, K4, K55, K64, KI11, KI14, KI2021, KI21, KI3, KI43, KI44, KY21, KY226, KY228, L317, L578, M14, M162W, M37W, MEF156.55.2, MO17, MO18W, MO1W, MO24W, MO44, MO45, MO46, MOG, MP339, MS1334, MS153, MS71, MT42, N192, N28HT, N6, N7A, NC222, NC230, NC232, NC236, NC238, NC250, NC258, NC260, NC262, NC264, NC294, NC296, NC296A, NC298, NC300, NC302, NC304, NC306, NC310, NC314, NC318, NC320, NC324, NC326, NC328, NC33, NC336, NC338, NC342, NC344, NC346, NC348, NC350, NC352, NC354, NC356, NC358, NC360, NC362, NC364, NC366, NC368, ND246, OH40B, OH43E, OH603, OH7B, OS420, P39, PA762, PA875, PA880, PA91, R168, R177, R229, R4, SA24, SC357, SC55, SD44, SG1533, SG18, T232, T8, TX303, TZI10, TZI11, TZI16, TZI18, TZI25, TZI8, TZI9, U267Y, VA102, VA14, VA22, VA35, VA59, VA99, VAW6, W117HT, W153R, W182B, W64A, WD, 33.16, 38.11, X4226, X4722

PopulationB

B73, MO17, 33.16, A188, A239, A619, A632, A634, A635, A641, A654, A661, A679, A680, A682, B103, B104, B109, B115, B14A, B37, B46, B52, B57, B64, B68, B73, B73HTRHM, B75, B76, B77, B79, B84, C103, C49A, CH701.30, CM105, CM174, CO125, DE.2, DE1, DE811, EP1, H105W, H49, H84, H91, H95, H99, HP301, IL101, IL14H, K148, KY226, M14, MEF156.55.2, MO44, MO45, MO46, MO47, MS1334, MS153, MS71, N192, N28HT, N6, NC262, NC264, NC294, NC306, NC310, NC314, NC324, NC326, NC328, NC342, NC364, ND246, OH43, OH43E,

OS420, P39, PA762, PA875, PA880, PA91, R168, R177, R4, SD40, SD44, SG18, VA102, VA14, VA17, VA22, VA35, VA85, VA99, W182B, W22, W64A, WF9, YU796.NS.
