# Supplementary material for: The Pattern and Distribution of Deleterious Mutations in Maize
Source: G3 (Bethesda). 2013 Nov 26;4(1):163–71. doi: 10.1534/g3.113.008870 (PMC3887532; doi:10.1534/g3.113.008870)
Supplement: Supporting Information [file supp_g3.113.008870_FileS2.pdf]

File S2

List of genomes used for reciprocal BLAST

Aquilegia coerulea, Arabidopsis lyrata, Arabidopsis thaliana, Brachypodium distachyon, Brassica rapa, Capsella rubella, Carica papaya, Chlamydomonas reinhardtii, Citrus clementina, Citrus sinensis, Cucumis sativus, Eucalyptus grandis, Glycine max, Linum usitatissimum, Malus domestica, Manihot esculenta, Medicago truncatula, Mimulus guttatus, Oryza sativa, Panicum virgatum, Phaseolus vulgaris, Physcomitrella patens, Populus trichocarpa, Prunus persica, Ricinus communis, Selaginella moellendorffii, Setaria italica, Sorghum bicolor, Thellunigiella halophila, Vitis vinifera, Volvox carteri.
