# Supplementary material for: The Pattern and Distribution of Deleterious Mutations in Maize
Source: G3 (Bethesda). 2013 Nov 26;4(1):163–71. doi: 10.1534/g3.113.008870 (PMC3887532; doi:10.1534/g3.113.008870)
Supplement: Supporting Information [file supp_g3.113.008870_TableS1.pdf]

Table S 1: List of Analyzed traits

Traits	Abbreviation	Populations
Days to tasseling	DTT	A
Tassel length (cm)	TSLLEN	A
Tassel branch count	TSLBCHCNT	A
Tassel angle	TSANG	A
Plant height (cm)	PLTHT	A & B
Upper leaf angle	UPLFANG	A
Leaf width (cm)	LFWDT	A
Leaf length (cm)	LFLEN	A
Kernel height	KNLHGT	A
Kernel weight	TOTKNLWT	A
Stem puncture resistance (kg/section)	RPR	A
Plant yield (g/plant)	PLTYLD	A
Ear length (cm)	EARLGH	A & B
10 kernel weight (g)	10KWT	A
Cob diameter (cm)	COBDIA	A & B
Cob weight (g)	COBWT	A & B
Seed number per ear	SEEDNB	B
