# Supplementary material for: The Pattern and Distribution of Deleterious Mutations in Maize
Source: G3 (Bethesda). 2013 Nov 26;4(1):163–71. doi: 10.1534/g3.113.008870 (PMC3887532; doi:10.1534/g3.113.008870)
Supplement: Supporting Information [file supp_g3.113.008870_TableS2.pdf]

Table S 2: Detailed results of the prediction of deleterious amino acids with MAPP, using the different gene sets, and with SIFT

	MAPP			SIFT
Gene sets	BLASTX	Reciprocal BLAST	Syntenic genes	PSI-BLAST
Total a.a. positions with predictions	7,746,638	5,570,035	6,869,010	11,906,167
Total number of genes	20,348	11,918	17,957	31,843
Number of positions covered by SNPs	74,909	52,283	72,562	112,326
Number of genes covered by SNPs	12,561	8,553	12,615	19,145
Monomorphic tolerated	39,009	25,270	39,300	58,685
Monomorphic not tolerated*	144	3470	14	387
Polymorphic tolerated	18,379	10,753	17,792	42,606
Polymorphic not tolerated*	17,377	12,790	15456	10,648

*Includes premature stop codons
