# Supplementary material for: The Pattern and Distribution of Deleterious Mutations in Maize
Source: G3 (Bethesda). 2013 Nov 26;4(1):163–71. doi: 10.1534/g3.113.008870 (PMC3887532; doi:10.1534/g3.113.008870)
Supplement: Supporting Information [file supp_g3.113.008870_TableS3.pdf]

Table S 3: Comparion of the results of MAPP predictions with the different gene sets.

Gene sets	BLASTX	Reciprocal BLAST	Syntenic genes
BLASTX	-	80.1%	78.2%
Reciprocal BLAST	38,054 (6,169)	-	79.8%
Syntenic genes	45,412 (7,745)	32,222 (5,488)	-

The lower triangle indicates the number of amino acid positions predicted with two given gene sets and covered by GBS SNPs (number of genes between brackets); the upper triangle indicates the percentage of amino acids with the same predictions.
