# Supplementary material for: The Pattern and Distribution of Deleterious Mutations in Maize
Source: G3 (Bethesda). 2013 Nov 26;4(1):163–71. doi: 10.1534/g3.113.008870 (PMC3887532; doi:10.1534/g3.113.008870)
Supplement: Supporting Information [file supp_g3.113.008870_TableS4.pdf]

Table S 4: Total number of significant SNPs in genic regions (n) and fold enrichment (f) for SNPs with deleterious mutations in population B. Numbers marked with "*" are statistically significant (Fisher's exact test p-value< 0.05).

	Inbreds		BPH_B73		MPH_B73		BPH_Mo17		MPH_Mo17	
Traits	*n*	*f*	*n*	*f*	*n*	*f*	*n*	*f*	*n*	*f*
10KWT	310	0.77	404	1.17*	257	0.86	698	0.83	723	0.98
COBWT	313	0.62	941	1.15*	387	0.69	257	1.33	532	0.95
COBDIA	226	1.49	159	1.25*	236	1.06*	349	0.78	615	0.72
COBLEN	598	1.08	239	1.20*	97	0.24	280	1.08	140	0.92
SEEDWT	362	1.09	378	1.32*	118	1.23*	1043	0.92	1080	0.78
SEEDNB	373	0.99	320	0.86	251	0.92	348	1.06	454	0.82
PLTHT	505	1.02	261	0.89	143	1.45*	1022	1.08	156	1.16
