# Supplementary material for: The Pattern and Distribution of Deleterious Mutations in Maize
Source: G3 (Bethesda). 2013 Nov 26;4(1):163–71. doi: 10.1534/g3.113.008870 (PMC3887532; doi:10.1534/g3.113.008870)
Supplement: Supporting Information [file supp_g3.113.008870_TableS5.pdf]

Table S 5: Total number of genes with significant SNPs (n) and fold enrichment for genes with predicted deleterious SNPs (f) in population B. Numbers marked with "*" are statistically significant (Fisher's exact test p-value< 0.05).

	Inbreds		BPH_B73		MPH_B73		BPH_Mo17		MPH_Mo17	
Traits	*n*	*f*	*n*	*f*	*n*	*f*	*n*	*f*	*n*	*f*
10KWT	73	1.17	169	1.14	95	1.11	246	1.11	274	1.11
COBWT	71	1.13	316	1.08	128	1.04	94	1.10	204	1.10
COBDIA	81	1.07	57	1.08	86	1.11	134	1.03	234	1.14
COBLEN	203	1.09	89	1.24	30	1.17	110	1.17	51	1.21
SEEDWT	138	1.10	146	1.14	50	0.97	371	1.09	389	1.09
SEEDNB	106	1.15	128	1.13	116	0.98	130	1.12	166	1.09
PLTHT	169	1.15	112	1.09	65	1.13	348	1.15	65	1.15
